# Modulated Laser Cladding of Implant-Type Coatings by Bovine-Bone-Derived Hydroxyapatite Powder Injection on Ti6Al4V Substrates—Part I: Fabrication and Physico-Chemical Characterization

**DOI:** 10.3390/ma15227971

**Published:** 2022-11-11

**Authors:** Aura-Cătălina Mocanu, Florin Miculescu, George E. Stan, Iuliana Pasuk, Teddy Tite, Alexandru Pascu, Tudor Mihai Butte, Lucian-Toma Ciocan

**Affiliations:** 1 Department of Metallic Materials Science, Physical Metallurgy, University Politehnica of Bucharest, 313 Splaiul Independentei, J Building, RO-060042 Bucharest, Romania; mcn_aura@hotmail.com (A.-C.M.); butte.tudor@yahoo.com (T.M.B.); 2 National Institute of Materials Physics, 405A Atomistilor Street, RO-077125 Măgurele, Romania; george_stan@infim.ro (G.E.S.); iuliana.pasuk@infim.ro (I.P.); teddy.tite@infim.ro (T.T.); 3 Department of Materials Engineering and Welding, University Transilvania of Brasov, 29 Eroilor Blvd., RO-500036 Brasov, Romania; alexandru.pascu@unitbv.ro; 4 Prosthetics Technology and Dental Materials Department, “Carol Davila” University of Medicine and Pharmacy, 37 Dionisie Lupu Street, RO-020022 Bucharest, Romania; tciocan@yahoo.com

**Keywords:** laser cladding, bioceramic coating, biological HA, powder injection, laser power

## Abstract

The surface physico-chemistry of metallic implants governs their successful long-term functionality for orthopedic and dentistry applications. Here, we investigated the feasibility of harmoniously combining two of the star materials currently employed in bone treatment/restoration, namely, calcium-phosphate-based bioceramics (in the form of coatings that have the capacity to enhance osseointegration) and titanium alloys (used as bulk implant materials due to their mechanical performance and lack of systemic toxicity). For the first time, bovine-bone-derived hydroxyapatite (BHA) was layered on top of Ti6Al4V substrates using powder injection laser cladding technology, and then subjected, in this first stage of the research, to an array of physical-chemical analyses. The laser processing set-up involved the conjoined modulation of the BHA-to-Ti ratio (100 wt.% and 50 wt.%) and beam power range (500–1000 W). As such, on each metallic substrate, several overlapped strips were produced and the external surface of the cladded coatings was further investigated. The morphological and compositional (SEM/EDS) evaluations exposed fully covered metallic surfaces with ceramic-based materials, without any fragmentation and with a strong metallurgical bond. The structural (XRD, micro-Raman) analyses showed the formation of calcium titanate as the main phase up to maximum 800 W, accompanied by partial BHA decomposition and the consequential advent of tetracalcium phosphate (markedly above 600 W), independent of the BHA ratio. In addition, the hydrophilic behavior of the coatings was outlined, being linked to the varied surface textures and phase dynamism that emerged due to laser power increment for both of the employed BHA ratios. Hence, this research delineates a series of optimal laser cladding technological parameters for the adequate deposition of bioceramic layers with customized functionality.

## 1. Introduction

Currently, effective and eco-friendly technologies are favored for sustainably producing the materials required to meet the ever-growing needs of today’s society. The inability to swiftly provide biomedical solutions with unique (personalized) specifications based on the type of application presently poses important challenges for the manufacturing sector. Moreover, the prevalence of dental and orthopedic ailments has grown due to the increase in life expectancy of the population, as has the usage of endo-osseous implants [1,2,3]. Accordingly, studies show that around 20% of orthopedic implants require reintervention, with aseptic loosening and implant infections acting as the two leading causes of implant failure [4,5,6]. As a result, research studies are carried out worldwide in search of solutions capable of generating and enhancing the overall functionality, longevity, and biological response of metallic implants. The primary function of bone-regenerative implants is to promote osseointegration and to create a host-friendly environment for human cells, as well as preventing bacterial colonization [4,7,8,9,10].

In the last five decades, several sectors, including the biomedical sector, have experienced significant progress thanks to the industrial implementation of titanium (Ti) and its alloys [11]. Historically, stainless steel and cobalt-chrome alloys have been employed and preferred by surgeons; however, due to comparable or improved mechanical and biological responses, and lower mass density, titanium and its alloys have now superseded them [2]. However, their use in difficult conditions, under extreme wear and friction, is severely constrained due to their insufficient tribological characteristics; as such, they cannot always be used directly [12]. Nevertheless, some titanium super-alloys, such as Ti6Al4V or Ti6Al7Nb, constitute the prevalent metallic biomaterials employed in the development of a large array of biomedical applications (e.g., surgical instruments, orthopedic and dental implants, cardiovascular devices, or fasteners [2,11]), due to their superior biocompatibility and mechanical performances, but they are not without drawbacks (e.g., they can fail due to corrosion processes when in contact with biological fluids) [11,12,13,14].

In order to circumvent the aforementioned issues and to endow improved in situ anchorage and osteogenic capabilities to Ti implant surfaces, many physical and chemical techniques have been tested over the years (e.g., surface roughening, acid etching, thermal oxidation, and electrochemical treatments, alloying, laser cladding, laser surface remelting, surface coating by chemical methods, physical vapor deposition technologies, or anodization) [6,11,12,15,16,17,18,19]. During the last decade, innovations in osseointegrative implants have been concentrated on topological and physico-chemical surface modifications. Different surface properties, including chemical composition, morphology, topography, roughness, and wettability, are crucial during the early stages of bone healing, and influence the overall functionality and success of the implant [6,15,20].

One of the most popular heat-energy-dependent non-conventional surface modification methods for metallic surface modification is laser cladding [11]. In laser cladding, a defocused laser beam is utilized as a heating source, and, as a result, the preplaced or synchronously fed/injected powders, as well as a thin layer of the substrate, melt and solidify quickly, producing a metallurgical bond [7,11,21]. Outstanding metallurgical adhesion to the substrate, precision, and minimal dilution are desired and achieved by laser cladding, an improvement compared to most conventional methods [22,23]. The coatings thus obtained are generally characterized by a homogenous fine-grained microstructure and relatively excellent wear resistance and hardness [7,23]. Moreover, the purposefully designed and controlled microstructures of such coatings can improve the bone–implant interface strength [7]. 

In search for better wear resistance, high temperature oxidation resistance, and superior biocompatibility, several authors have devoted their efforts to the laser cladding of ceramic materials on titanium-based materials [7,21,24]. The majority of experimental research has been concentrated on the optimization of laser-process parameters, cladding material design (diluted or doped) [25,26,27], and stratified-layer architectures [12,13,16,28,29]. Although it is necessary for metallurgical bonding, one of the key problems is reducing the dilution and the thermal expansion mismatch by selecting the adequate process parameters [12,21,24]. Another key parameter relates to the laser power that significantly affects the creation of the cladded layer and the depth of penetration—the higher the laser power, the higher the diffusion of the substrate material into the powder bed, leading to a decrease in the coating quality [11]. 

In order to combine bioactivity with mechanical strength, bioceramics can be used as coatings on bioinert metallic substrates [14,24]. The osseointegration of a metal component is always challenging. Calcium-phosphate-based materials (CaPs), most frequently hydroxyapatite (HA), are applied as coatings to metal surfaces due to their physiologically (e.g., chemically, structurally, and biologically) comparable properties to the natural bone mineral component [13,14,30,31,32,33,34]. Bioceramic HA-based materials may be formed by laser cladding on Ti substrate either directly, by using an already synthesized material (from synthetic or natural resources [14,16,26,35,36,37,38]) as a source material, or indirectly, by utilizing two sets of materials (one Ca-rich material and one P-rich material) and subjecting them to a reactive deposition process [7,14]. When favored by the laser processing parameters, the formation of calcium titanate (CaTiO_3_) and tetracalcium phosphate (TTCP) as additional phases can occur, preferably with the preservation of part of the HA phase. This is viewed as proof of atomic inter-diffusion between the HA-layer and the Ti alloy substrate, and it is believed to be beneficial for improving a coating’s adherence, as well as for the rapid fixation and development of new bone on the metallic surface [16,21,39,40].

In this study, we explore for the first time the combined modulation of two governing parameters for laser cladding by powder injection, namely, the ratio of the HA coating material and the laser beam power, and their significant influence on the final physico-chemical and surface features of the Ti implants. Furthermore, our research design targets the novel implementation of a biogenic HA extracted from bovine bones (BHA), which is thermally stable at high temperatures (1000–1200 °C) [41,42,43], and thus more suitable for laser cladding processing. In fact, the predilection of synthetic HA to decompose into tricalcium phosphates—readily soluble phases [1,16]—was reported to induce the coating delamination and subsequent leaching of ions from the metallic substrate [1,24,44].

Sample sets prepared using non-diluted (100 wt% BHA) and diluted (50 wt.% BHA and 50 wt.% Ti) bioceramic materials and four gradually incremented laser powers (500–1000 W) were subjected to a comprehensive investigation focusing on the compositional (EDS), structural (XRD, micro-Raman), morphological (SEM), and surface wettability (contact angle and surface free energy) features. The proposed experimental design outlined a sustainable and tunable procedure/technology with delineated optimal processing parameters for good quality bioceramic coating manufacturing by laser cladding.

## 2. Materials and Methods

### 2.1. Sample Preparation

The biological hydroxyapatite (BHA) powder was synthesized from bovine bone precursors by an optimized and reproducible technology, as previously reported in detail in refs. [41,45], based on successively applied thermal treatments, followed by grounding and granulometric sieving. Half of the BHA powder batch with particle sizes <40 μm was used as is, and the other half was further mixed with pure titanium powder (Merck KGaA, Darmstadt, Germany) by mechanical homogenization at 50 rpm for 1 h (Inversina, Bioengineering AG, Zürich, Switzerland), at a BHA/Ti wt.% ratio of 50/50. The sample codification denotes the BHA ratio, 100% BHA, and 50% BHA, respectively. The prepared ceramic-based materials were kept in sterile glass dishes.

Disks (diameter, Φ = 50 mm; height, h = 5 mm) cut from the same Ti6Al4V bar were used as substrates for the ceramic coating deposition. Several parallel channels (width, w = 5 mm; depth, d = 2 mm) were further milled off, followed by filing, throughout the entire diameter of the disk-shaped samples. Three identical metallic samples were prepared for each ceramic-based material for the validation of results. All metallic disks were then subjected to a stress-relief-annealing treatment [46], performed at 540 °C for 2 h. 

Furthermore, the laser cladding process was carried out along all machined channels of the metallic disk-shaped substrates by direct coaxial injection of the ceramic powder, using a Rofin YC3300 laser (1064 nm, 3.3 kW) and a Rofin coaxial injection unit model Coax 8 (Rofin-Sinar, Plymouth, MI, USA) manipulated by a 6 axes IRB 4400 robot (ABB ASEA Brown Boveri, Zürich, Switzerland). The ceramic powder was supplied to the cladding head under argon gas flow (10 L/min). The optimum parameters for laser cladding by powder injection were established as: a powder feed rate of 6 g/min and a scanning speed of 4.3 mm/s [47]. In order to obtain the lowest possible dilution with the substrate, a defocused 2.5 mm laser beam was used [22].

Partially overlapped strips were induced with four laser beam powers (500, 600, 800, and 1000 W) for each type of sample. After completing the laser cladding process, only the external surfaces of the coated areas were subjected to physico-chemical investigations.

### 2.2. Sample Characterization

(1) The chemical composition was determined by energy dispersive X-ray spectroscopy (EDS) using a microanalysis system (EDAX Sapphire UTW, 128 eV resolution, AMETEK Inc., Berwyn, PA, USA) attached to the scanning electron microscope, in five randomly selected areas of each specimen.
(2) The crystalline status of the laser-cladded samples was investigated by X-ray diffraction (XRD) in symmetric (θ–θ) geometry using a Rigaku SmartLab 3 kW system (Rigaku Corporation, Tokyo, Japan) with CuK_α_ radiation (λ = 1.5418 Å). The diffractometer was equipped with an HyPix-3000 detector, operated in 1D mode. The XRD measurements were conducted in parallel beam setting, in the 2θ range 20–90° with a step size of 0.02° and speed of 3 degrees/min.
(3) In situ micro-Raman experiments were carried out using a LabRAM HR Evolution confocal spectrometer (Horiba Jobin-Yvon, Edison, NJ, USA) to analyze the structural properties of the samples based on their vibrational signatures. A He-Cd laser with a wavelength of 325 nm was focalized in backscattering geometry on the surface of the samples with a Thorlabs LMU-40× objective (Thorlabs Inc., Newton, NJ, USA). The Raman spectra were calibrated using the Rayleigh (0 cm^–1^) and silicon (520.7 cm^–1^) standard bands. The scattered light was recorded at different laser powers in the 150–2000 cm^–1^ spectral range using 2400 lines/mm diffraction grating. The spectral resolution was ~1 cm^–1^.
(4) The morphological characteristics were evaluated by scanning electron microscopy (SEM), using a Phillips XL 30 ESEM TMP microscope (FEI/Phillips, Hillsboro, OR, USA). The acquisition of the micrographs was conducted at an acceleration voltage of 25 kV and a working distance of 10 mm, in five randomly chosen areas.
(5) The wettability features were evaluated by water contact angle measurements using a Krüss Drop Shape Analyzer—DSA100 (A. Krüss Optronic GmbH, Hamburg, Germany). The experiments involved three wetting agents (water and ethylene glycol (EG) as polar agents, and diiodomethane (DIM) as a dispersive agent) and controlled ambient parameters (temperature of 20 ± 1 °C and room humidity of 45 ± 5%). The images were captured 1 s after the deposition of the wetting agent droplet. The results were processed with the ImageJ 1.50 software (National Institutes of Health, Bethesda, MD, USA) and an average of 5 determinations/sample were performed. The surface free energy was computed by the Owens, Wendt, Rabel, and Kaelble (OWRK) method [48].

## 3. Results and Discussion

### 3.1. Compositional Evaluation

**Figure 1:**
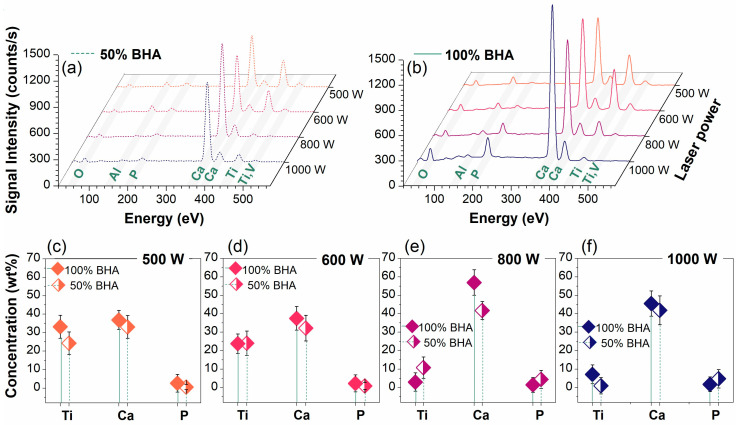
Characteristic EDS spectra collected for the laser-cladded samples deposited using BHA powder contents of (**a**) 50 and (**b**) 100 wt.%. Elemental concentration evolution function of (**c**–**f**) the BHA ratio at the four laser beam powers: (**c**) 500 W; (**d**) 600 W; (**e**) 800 W; and (**f**) 1000 W.

The chemical composition results recorded for the laser cladded samples are presented in Figure 1 and Figure S1. The evolution of the EDS spectra denoted a clear influence of both the BHA ratio and the laser power parameters. The laser cladding process usually generates a thermo-chemical reaction by melting the coating material on the metallic substrate surface. As such, the metallic bonding, also known as the ‘dilution phenomenon’, appears and is further favored by the input laser power and, implicitly, by the resultant high temperature and heating rate on various areas of the sample [16,22,49].

Only the constituting elements (i.e., Ca, P, O, Ti, Al, V) of the ceramic (BHA) and metallic (Ti6Al4V) starting materials involved in laser the cladding processing were evidenced by EDS. The formation of the ceramic cladded layer under the influence of laser power led to varied signal intensities for all identified elements, function of the BHA ratio (i.e., slightly higher intensities for 100 wt.% BHA samples were observed). However, the semi-quantitative showed a quasi-linear increase in Ca and P concentrations, with the increase in the laser power above 600 W, at the expense of Ti and V. Moreover, the Al content was gradually reduced up to the maximum laser power, while the O concentration was preserved at low laser powers and significantly increased at 1000 W, suggesting the possible formation of additional compounds (bright gray areas marked 3 in Figure S1). As presented in Figure S1, this variation in the samples’ chemical composition was also documented for the three different gray areas spotted in the micrographs (see SEM results below).

The give-and-take behavior of the chemical elements was also reflected in the calculated Ca/P ratios, which ranged between 13.93–37.47 and 9.37–39.04 for samples deposited using BHA contents of 50 and 100 wt.%, respectively, and which were, overall, higher than the reported values [45,50].

### 3.2. XRD Investigations

**Figure 2:**
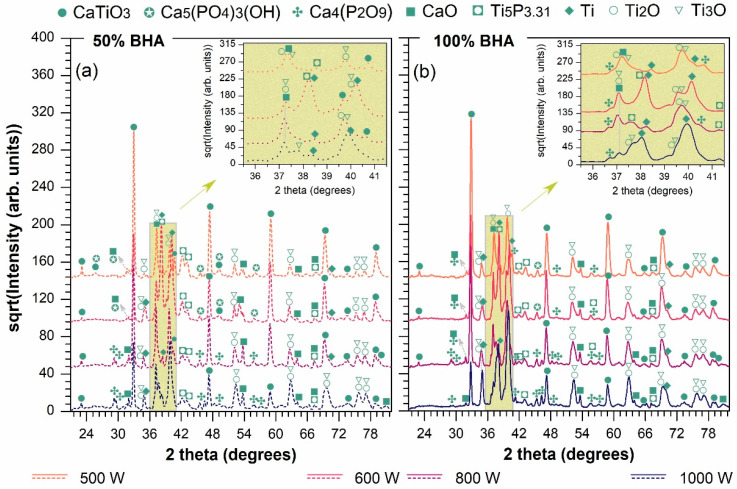
XRD patterns collected for the laser-cladded samples deposited using BHA powder contents of (**a**) 50 and (**b**) 100 wt.% at the four beam powers.

The XRD patterns of all laser-cladded samples prepared using the powder injection method are plotted in Figure 2. The formation of diverse compounds, as already suggested by the chemical composition results (see EDS results above), was further supported by the crystalline phase composition evidenced by XRD. This phenomenon takes place when molten pools are created at the substrate surface and the subsequent rapid cooling in the air atmosphere interferes with the uniform homogenization of the input materials, acting as a major factor for the prospected features of the cladded layers [49].

Here, the conjoint modulation of the key processing parameters favored the formation of a complex assortment of crystalline phases specific to/derived from the metallic substrate, the ceramic coating material, or their blending. Such compounds were found for both types of sample prepared using different BHA/Ti ratios. Hence, the characteristic intense peaks, which have an evolution inversely related to the laser power increment, were found to belong to calcium titanate (CaTiO_3_, orthorhombic—ICDD-PDF4: 00-022-0153); this formed as the dominant phase in the case of all samples, except those prepared at the 1000 W beam power. The higher the beam power applied, the higher the generated temperature was. Consequently, in such conditions, the interaction between the BHA powder and the titanium dioxide, naturally occurring on the substrate surface, led to the development of the CaTiO_3_ phase, known to be biocompatible [16,21,39,49]. When subjected to repeated high temperatures, the partial dehydroxylation of the bioceramic material can be induced, leading to the advent of small amounts of calcium oxide (CaO, cubic—ICDD-PDF4: 01-080-7710); this result is in good agreement with the literature [51].

As stated before, all laser cladded areas were subject to multiple dynamic reactions, all of which are impacted by the possible modulation of the processing temperature and convertible dilution of the input materials [16,49]. It is important to note that the XRD data confirmed, for both types of sample, that part of the original BHA source compound (indexed with a HA-type phase, Ca_5_(PO_4_)_3_(OH), ICDD-PDF4: 04-010-6314) was preserved after the laser processing at beam powers of 500 and 600 W. In tandem, the tetracalcium diphosphate (TTCP, Ca_4_(P_2_O_9_), monoclinic—ICDD-PDF4: 01-076-7650) emerged as a direct result of the HA decomposition at high temperatures [30,52,53,54]. At increased beam powers, the BHA phase vanished and only the derived biocompatible TTCP phase [54] could be clearly outlined. The existence of TTCP in the hydroxyapatite-based laser-cladded areas is known to positively/effectively modify the in situ solubility and resorbability rates of the coatings [53]. These findings testify to the partial consumption of BHA and the initial formation of CaTiO_3_, functions of the stability and material recirculation in the melting zone [49], regardless of the beam power and BHA input mass. 

Another adjacent detected event refers to the appearance and maintenance of the titanium phosphide phase (Ti_5_P_3.31_, hexagonal—ICDD-PDF4: 04-003-1940), evidenced by individual specific peaks and/or superimposed to those of the CaO and TTCP phases, in the case of all investigated samples. However, even though the Ti_5_P_3.31_ phase is known to generally occur at elevated temperatures [24], its peak intensities appeared to decrease with the increase in the beam power. Furthermore, in a laser-power-dependent process, the auxiliary titanium oxide phases (Ti_2_O, hexagonal—ICDD-PDF4: 04-006-8712 and Ti_3_O, hexagonal—ICDD-PDF4: 04-005-4376) evolved to the detriment of all the above mentioned compounds—this becomes clearly visible starting with the 600 W beam power. At the highest beam power (i.e., 1000 W), due to the rapid instances of dilution with the substrate material and the concomitant replacement/consumption of the initial ceramic component, sharp peaks indexed to pure titanium (Ti, hexagonal—ICDD-PDF4: 01-086-2608) emerged and developed as the main phase, being more pronounced for the samples prepared using 100 wt.% BHA. At lower beam powers, the Ti peaks had lower intensities, being overlapped by the characteristic peaks of the titanium oxides, TTCP and CaTiO_3_. This flow of events was prominently observed when comparing the two sample sets in the areas with clustered peaks, outlined as insets in Figure 2. One can also stress that, in contrast to the 100 wt.% BHA samples, the 50 wt.% BHA samples yielded the TTCP phase only at the 800 and 1000 W beam powers. As such, it is suggested that the modulation of the ceramic powder dilution and the laser power can be used as tools to derive advantageous final compositions for the laser cladded areas.

### 3.3. Raman Spectroscopy Analysis

**Figure 3:**
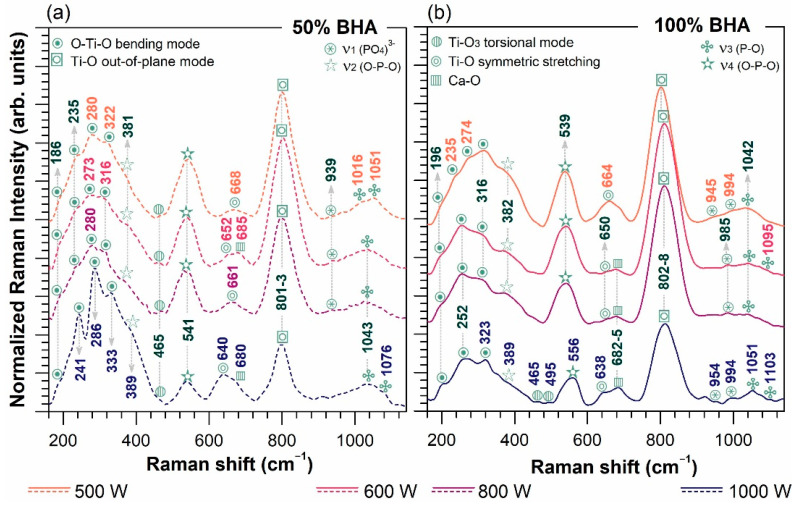
Raman spectra collected for the laser-cladded samples deposited using BHA powder contents of (**a**) 50 and (**b**) 100 wt.% at the four beam powers.

The Raman spectra of all laser-cladded areas prepared with various BHA ratios are comparatively presented in Figure 3. The depicted spectroscopic results endorse the XRD findings (see XRD results above). The envelopes of the specific Raman modes in the 150–1150 cm^–1^ region of interest were identified for all samples, however, presenting changes function of BHA content and laser beam power. The presence of CaTiO_3_ was confirmed by four Raman active modes with quasi-similar peak evolution:

▪ The O–Ti–O bending mode, visible at 196, 235–252, 274, 316–323 cm^–1^ for samples prepared with 100 wt.% BHA and at slightly shifted frequencies of 186, 235–241, 273–286, 316–333 cm^–1^ [6,55,56,57,58] for samples deposited using 50 wt.% BHA content;
▪ The Ti–O_3_ torsional mode, found as low-intensity single or double shoulder(s), only for samples obtained at 1000 W beam power and 100 wt.% BHA (465, 495 cm^–1^) and for all of those prepared using 50 wt.% BHA (465 cm^–1^) [55,56,57,59];
▪ The Ti–O symmetric stretching mode, formed as a single peak for samples obtained at 500 W beam power with 100 wt.% BHA (664 cm^–1^) and 50 wt.% BHA (668 cm^–1^), and as a split-peak under increased laser power, at 638–650 cm^–1^ and 640–661 cm^–1^ for non-diluted and diluted BHA, respectively [55,56,57,59,60]. The second order contribution for this peak was attributed to the isolated CaO Raman mode (680–685 cm^–1^) as a result of BHA decomposition, in agreement with some previous reports [61] and the XRD data presented within;
▪ The Ti–O out-of-plane mode, evidenced as maximum frequency bands at 802–808 cm^–1^ (100 wt.% BHA) and 801–803 cm^–1^ (50 wt.% BHA) [60,62]. As a laser-power-dependent advent in the 500–600 W range, the progressively elevated intensities of the peaks exposed the CaTiO_3_ phase tendency to become more stable when high temperatures are reached [49]. However, at a higher beam power, the development was inversely switched, endorsing the XRD findings in this respect.

As expected, the formation of the additional TTCP phase during the HA decomposition was also exposed by the detection of characteristic and well-defined Raman bands, drastically reduced in intensity at maximum beam power, originating mostly to a factor group splitting of several bands (i.e., 420, 567, 938, 1017 cm^−1^) [52,54]:

▪ the ν_2_ and ν_4_ bending modes of the O–P–O functional group identified at 382–389 cm^−1^ and 539–556 cm^−1^, and 381–389 cm^−1^ and 541 cm^−1^, for the 100 wt.% BHA and 50 wt.% BHA samples, respectively;
▪ the ν_1_ bending mode and ν_3_ stretching mode of the orthophosphate functional groups, encountered at 1042–1051 cm^−1^ and 1095–1103 cm^−1^, and 1016 cm^−1^, 1043–1051 cm^−1^ and 1076 cm^−1^, for the 100 wt.% BHA and 50 wt.% BHA samples, respectively. However, the emergence of the typical vibrational bands of the ν_3_ stretching (965–1100 cm^−1^) mode of the (PO_4_)^3−^ group, arising from the BHA phase, are overlapped on the same spectral region [13,63,64].

### 3.4. Morphological Evaluation

**Figure 4:**
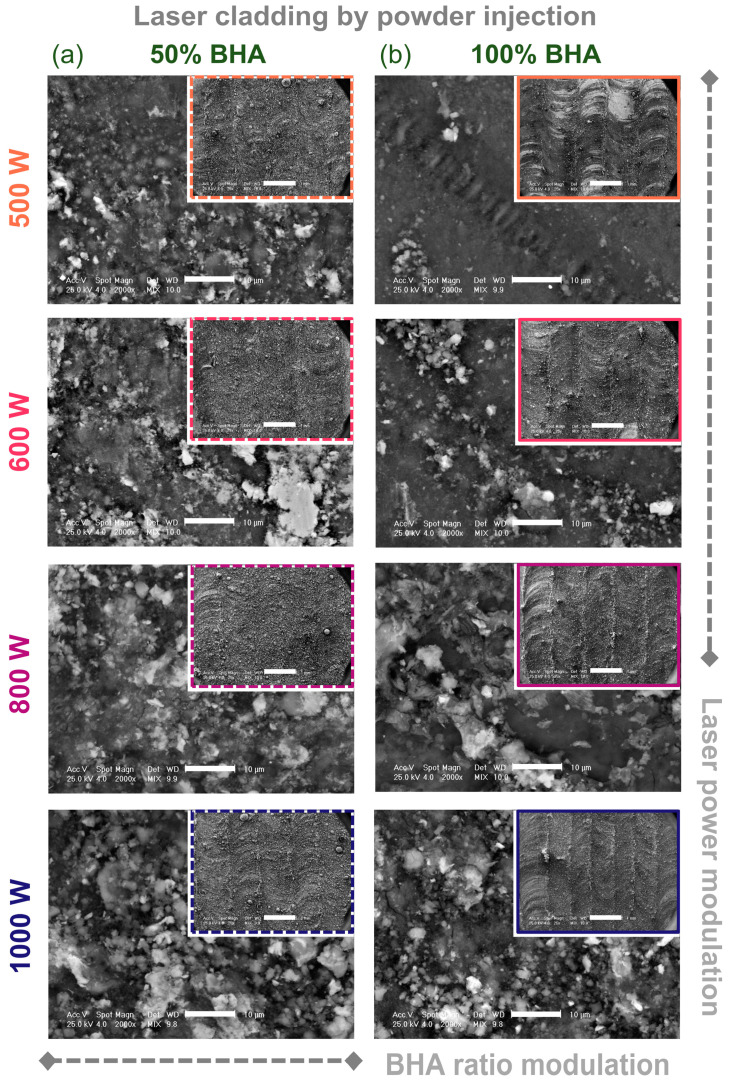
SEM morphological evaluation of the laser cladded samples deposited using BHA powder contents of (**a**) 50 and (**b**) 100 wt.% at the four beam powers. Scale bar: main image—10 μm, inset—1 mm.

The surface morphology of the laser-cladded samples are displayed in Figure 4. For a general evaluation, the micrographs acquired at low magnification are presented as insets, while the main images reveal the high-magnification detailed topography of the coatings. Supplementary details regarding the morphology in cross-sectional views for all samples can be found in Figure S1.

As presented before, for the laser cladding to take place, the laser beam power should be high enough to endorse the concomitant melting and mixing of the injected powder and a small amount of the substrate material, followed by the rapid cooling stage [16,21,39]. As such, the gradual passing of the laser beam along the straight-machined channels and in an alternate back-and-forth manner from one channel to another parallel one led to the appearance of regularly distanced circular marks/grooves and linear overlapping strips, similar to a weld bead, which were clearly visible for the samples prepared using 100 wt.% BHA. The prominent linear surfaces (as seen in the inset images), which may be mistaken at a first glance with cracks, actually represent the overlapping regions of each two-by-two cladded strip. The BHA powder dilution prior to laser cladding generated a more uniform yet rougher general aspect of the cladded layers. Nevertheless, in the case of all samples, no areas remained uncovered by the newly formed ceramic-based layer and no fractures were induced, regardless of the BHA ratio employed or the beam power. These findings are also backed by the morphology in the cross-section view for all samples, as presented in Figure S1. Moreover, the supplementary results advocated for the strong metallurgical bond induced between the two materials during laser cladding, which is desired for curtailed failure instances of the metallic implants and for general behavior that is compatible with the biomedical applications [39]. Here, one can easily note that no fragmentation or delamination of the ceramic layer from the substrate occurred during the laser cladding processing at laser beam powers of 500–800 W. The only apparent fragmentation was isolated for samples with a 50 wt.% BHA ratio at the interface of the cladded layer and the immediately adjacent heat-affected zone, and was favored by the maximum beam power (1000 W).

Further, the detailed micrographs (Figure 4) exposed the significant influence of the two modulated parameters through the development of the cladded layers on two connected levels from the substrate. The proximal (first) level fully encased the ceramic polyhedral particles in a uniform and homogenous manner, as seen only for the sample prepared with 100 wt.% BHA at 500 W (dark gray areas marked 1 in Figure S1). For all the other samples, these areas were spread randomly on the surface and can be depicted as valleys from the second formed level. This level is shaped during the quick solidification phase that favors the ceramic particle-to-particle attachment and the nucleation of free-form aggregates/conglomerates [39] that are strongly embedded in the cladded layer. As such, the higher the laser power (and, implicitly, the temperature), the higher the generated thermal expansion and local tensile stress coefficients are between the two materials (the metallic substrate and injected ceramic powder) [16,24]. Therefore, this parameter governed the entire cladding process by powder injection.

The inception of aggregate formation is delineated for all samples as the lighter gray zones progressively grow in number and size, starting with 500 W beam power up to the maximum power level. At this stage, a significant amount of BHA particles remained in an incomplete homogenization state, regardless of the initial BHA content, and in the end this created rougher surfaces for the cladded layers (light gray areas marked 2 in Figure S1). In this regard, the scientific literature points towards the beneficial effect of surface roughness for bone cell proliferation and, eventually, the new bone structure’s ability to anchor to the implant’s surface [2,4,49]. This translates to improved stability and osteogenic responses, prerequisites for both orthopedic and dental applications. 

Overall, compared to conventional coating techniques, the morphological findings demonstrate the efficiency and ability of the laser cladding process by powder injection to produce a solid metallurgical connection between the starting materials, independent of the ceramic powder content or the laser power [24].

### 3.5. Contact Angle Measurements

The wettability behavior and surface free energy (SFE) were further investigated as two important triggers for cell adhesion, proliferation and differentiation responses and growth factor adsorption on the implant’s surface [65,66,67,68,69].

**Figure 5:**
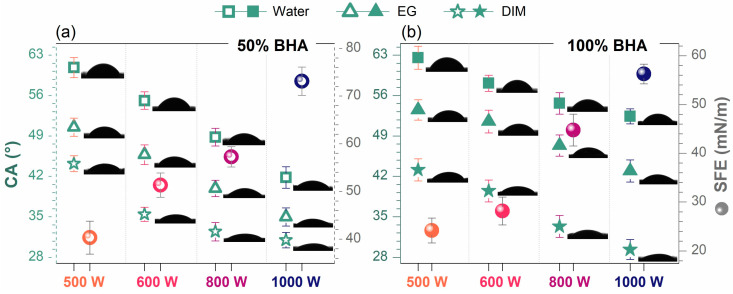
Contact angle measurements with three wetting agents (water, diiodomethane (DIM), and ethylene glycol (EG)), and Surface Free Energy (SFE) determined by the OWRK method, of the laser-cladded samples deposited using BHA powder contents of (**a**) 50 and (**b**) 100 wt.% at the four beam powers.

All cladded samples exhibited a linear decrease in CA values (for all testing liquids) with the beam power increment, independent of the BHA powder content (presented in Figure 5). The recorded CA values were found in the ranges of ~62–52° (water), ~43–29° (DIM) and ~53–42° (EG), and ~60–41° (water), ~44–30° (DIM), and ~50–34° (EG) for the 100 wt.% BHA and 50 wt.% BHA samples, respectively. This hydrophilic behavior (CA < 90°) elicited by all samples is induced by both the phase composition and morphological features of the cladded samples. The preservation of the hydrophilic BHA phase and the development of additional biocompatible yet hydrophilic phases, such as TTCP and CaTiO_3_ (Figure 2), enabled an improved wettability [16,54,70] when using a beam power higher than 600 W. The global effect is further strengthened by the surface texture (Figure 4) of the cladded layers, induced by the modulation of both parameters, namely, the formation of micrometric aggregates, especially at high laser power, which explains and supports the overall lower CA values (accentuated for samples deposited using the 50 wt.% BHA powder content) [48,69].

The evolution of the SFE values (Figure 5) revealed a behavior in agreement with the gradual modulation of the laser beam power, for both BHA/Ti powder ratios. As expected, the increase of SFE trendline endorsed the high wettability of all samples. As the morphology was influenced by the enlargement of the aggregates and the formation of rougher surfaces due to BHA particles homogenization during the cladded layer development, the overall contact surface area is enlarged, which can be to the benefit of cells attachment, proliferation, growth and survival [24,71,72]. In this respect, complex cell-culture tests, encompassing not only cell viability and proliferation, but also the ability of such coatings to induce the differentiation of stem cells towards the osteogenic phenotype will be performed as a mandatory future step in our research plan.

## 4. Conclusions

This research study focused on a complex assessment of ceramic coatings developed on a Ti6Al4V substrate using laser cladding by powder injection with bovine-bone-derived hydroxyapatite. The simultaneous modulation of two key parameters, the BHA/Ti ratio and the laser beam power, had a decisive influence on the physico-chemical features of the cladded layers. A series of important conclusions stemmed from this study:

- The low beam power regime allows for the conservation of the BHA phase and the formation of the biocompatible CaTiO_3_ compound.
- The high beam power regimes entail high temperatures, and lead to the partial decomposition of BHA and the advent of the biocompatible and soluble TTCP phase.
- Regardless of the laser beam power, yet with a slight preference towards the samples prepared using the 50/50 wt.% BHA/Ti ratio, the appearance of Ti_5_P_3.31_ was also noticed, along with a series of Ti oxides and sub-oxides and CaO phases, at the concentrational expense of the ubiquitous CaTiO_3_ phase.
- The surface of the cladded layers was marked by the formation of circular marks/grooves and overlapping strips in the direction in which the laser beam passed, independent of the BHA/Ti ratio or the laser beam power. The detailed examination exposed varied surface textures due to the formation of aggregates that gradually increased in number and size above 600 W for the 100 wt.% BHA samples and independently of the laser power for the 50 wt.% BHA samples.
- As a direct consequence of the excessive beam power, some BHA particles were incompletely homogenized.
- Favorably, the phase composition and morphological features induced the overall hydrophilic behavior of all samples, which is required for future biomedical applications.
- The laser cladding by powder injection process was demonstrated to be highly effective in creating a strong metallurgical bond between the metallic substrate and the ceramic coating.

It is thus expected that, depending on the requirements of the specific biomedical application, the BHA/Ti powder ratio and the laser beam power can be engineered in a favorable manner to enable the generation of coatings with adequate physico-chemical features. In future research, both mechanical tests and complex in vitro assays will be further performed to infer the true functional potential of these types of coating solutions. 

## Data Availability

Not applicable.

## Supplementary Materials

The following supporting information can be downloaded at: https://www.mdpi.com/1996-1944/15/22/7971/s1, Figure S1: SEM morphological evaluation of the laser cladded samples deposited using BHA powder contents of (**a**) 50 and (**b**) 100 wt.% at the four beam powers. Main image: cross-section view. Inset images: surface top-view. Scale bar: 20 μm.
