# Supplementary material for: Modulated Laser Cladding of Implant-Type Coatings by Bovine-Bone-Derived Hydroxyapatite Powder Injection on Ti6Al4V Substrates—Part I: Fabrication and Physico-Chemical Characterization
Source: Materials (Basel). 2022 Nov 11;15(22):7971. doi: 10.3390/ma15227971 (PMC9695758; doi:10.3390/ma15227971)
Supplement: Supplementary file 1 [file materials-15-07971-s001.zip › materials-2005735-supplementary.pdf]

Supplementary materials

FIGURES

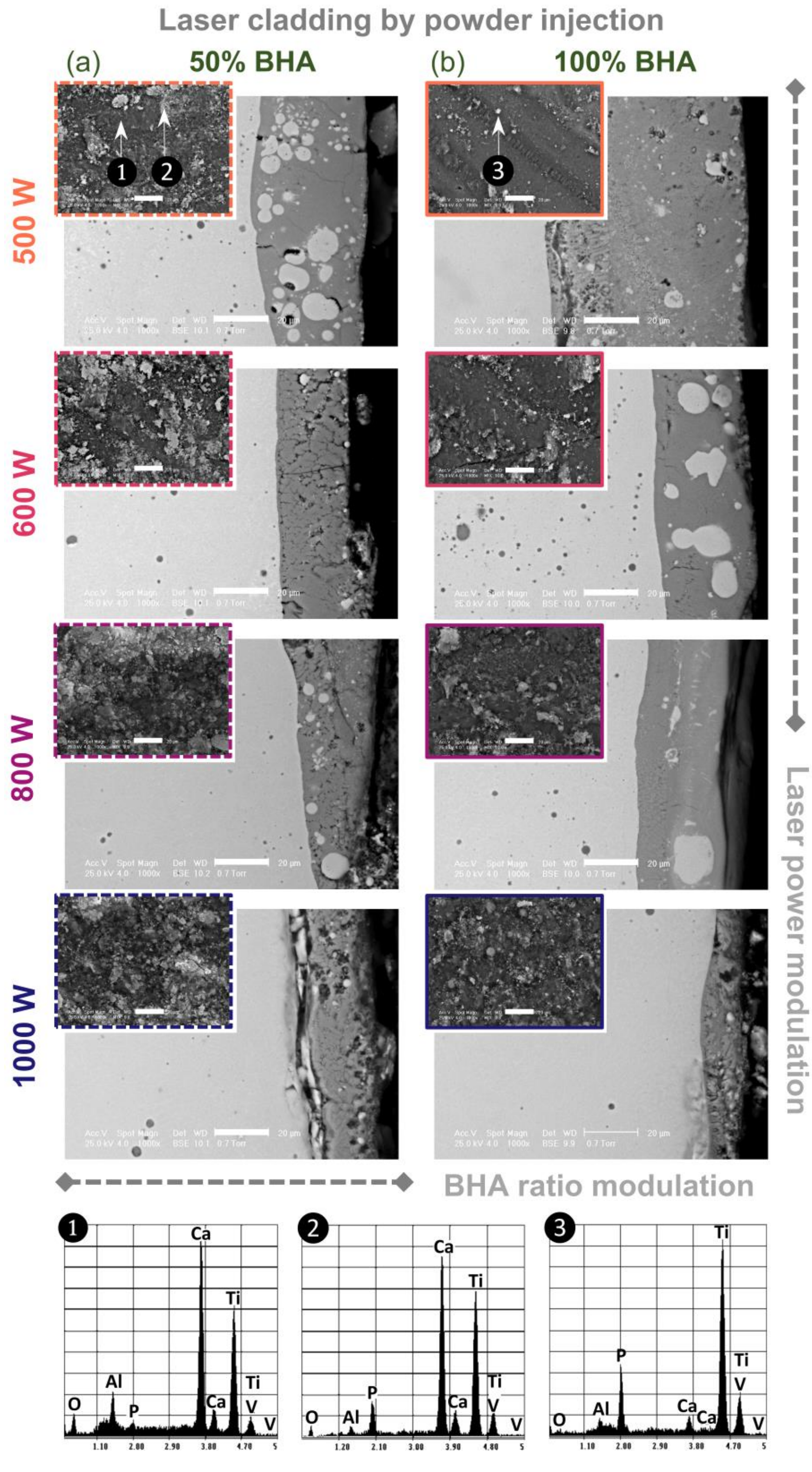

Figure S1. SEM morphological evaluation of the laser cladded samples deposited using BHA powder contents of (**a**) 50 and (**b**) 100 wt.% at the four beam powers. Main image: cross-section view. Inset images: surface top-view. Scale bar: 20 μm.
